# Loss of Humoral and Cellular Immunity to Invasive Nontyphoidal Salmonella during Current or Convalescent Plasmodium falciparum Infection in Malawian Children

**DOI:** 10.1128/CVI.00057-17

**Published:** 2017-07-05

**Authors:** Tonney S. Nyirenda, James T. Nyirenda, Dumizulu L. Tembo, Janet Storm, Queen Dube, Chisomo L. Msefula, Kondwani C. Jambo, Henry C. Mwandumba, Robert S. Heyderman, Melita A. Gordon, Wilson L. Mandala

**Affiliations:** a Pathology Department, College of Medicine, University of Malawi, Blantyre, Malawi; b Malawi Liverpool Wellcome Trust Clinical Research Programme, Blantyre, Malawi; c Liverpool School of Tropical Medicine, Liverpool, United Kingdom; d Department of Paediatrics and Child Health, Queen Elizabeth Central Hospital, Blantyre, Malawi; e Division of Infection and Immunity, University College London, London, United Kingdom; f Institute of Infection and Global Health, University of Liverpool, Liverpool, United Kingdom; g Biomedical Sciences Department, College of Medicine, University of Malawi, Blantyre, Malawi; University of Florida

**Keywords:** Salmonella, malaria, children, immunity, susceptibility

## Abstract

Invasive nontyphoidal Salmonella (iNTS) infections are commonly associated with Plasmodium falciparum infections, but the immunologic basis for this linkage is poorly understood. We hypothesized that P. falciparum infection compromises the humoral and cellular immunity of the host to NTS, which increases the susceptibility of the host to iNTS infection. We prospectively recruited children aged between 6 and 60 months at a Community Health Centre in Blantyre, Malawi, and allocated them to the following groups; febrile with uncomplicated malaria, febrile malaria negative, and nonfebrile malaria negative. Levels of Salmonella enterica serovar Typhimurium-specific serum bactericidal activity (SBA) and whole-blood bactericidal activity (WBBA), complement C3 deposition, and neutrophil respiratory burst activity (NRBA) were measured. Levels of SBA with respect to *S*. Typhimurium were reduced in febrile P. falciparum-infected children (median, −0.20 log10 [interquartile range {IQR}, −1.85, 0.32]) compared to nonfebrile malaria-negative children (median, −1.42 log10 [IQR, −2.0, −0.47], *P* = 0.052). In relation to SBA, C3 deposition on *S*. Typhimurium was significantly reduced in febrile P. falciparum-infected children (median, 7.5% [IQR, 4.1, 15.0]) compared to nonfebrile malaria-negative children (median, 29% [IQR, 11.8, 48.0], *P* = 0.048). WBBA with respect to *S*. Typhimurium was significantly reduced in febrile P. falciparum-infected children (median, 0.25 log10 [IQR, −0.73, 1.13], *P* = 0.0001) compared to nonfebrile malaria-negative children (median, −1.0 log10 [IQR, −1.68, −0.16]). In relation to WBBA, *S*. Typhimurium-specific NRBA was reduced in febrile P. falciparum-infected children (median, 8.8% [IQR, 3.7, 20], *P* = 0.0001) compared to nonfebrile malaria-negative children (median, 40.5% [IQR, 33, 65.8]). P. falciparum infection impairs humoral and cellular immunity to *S*. Typhimurium in children during malaria episodes, which may explain the increased risk of iNTS observed in children from settings of malaria endemicity. The mechanisms underlying humoral immunity impairment are incompletely understood and should be explored further.

## INTRODUCTION

Invasive nontyphoidal Salmonella (iNTS) infections, principally by Salmonella enterica serovar Typhimurium and S. enterica serovar Enteritidis, are estimated to cause over 2.1 million illnesses and 416,000 deaths per year (1). In settings of malaria endemicity, invasive NTS infections are commonly associated with current or convalescent episodes of malaria, in particular, severe malarial anemia (2, 3). Other factors associated with increased susceptibility to iNTS in children are immature immunity and malnutrition, while HIV infection is the driving force for iNTS susceptibility in adults (4, 5). About 6.5% of invasive bacterial infections (IBIs) occur in P. falciparum-infected children (6, 7); however, in view of the low sensitivity of blood cultures, P. falciparum infection might account for more than 50% of IBIs in children living in settings of malaria endemicity (8). Often, children are diagnosed and treated for malaria while IBI is left unattended, leading to poor health outcomes.

The association between malaria and iNTS was first reported in the 1920s (9). Biggs et al. recently reported that coinfections by iNTS and malaria were common in febrile pediatric in-patients from an area of high malaria transmission compared to those from an area of low malaria transmission in Tanzania (10). In contrast, *S*. Typhi bacteremia was uncommon in febrile pediatric in-patients from an area of high malaria transmission (10). In addition, the association between iNTS and malaria is observed in seasonal peaks during the rainy season (4, 5, 11, 12). However, the immunologic basis for increased numbers of iNTS cases in settings of malaria endemicity is not fully understood.

The link between NTS and malaria in humans and mice is extensively covered in the reviews by Uche et al. (13) and Takem et al. (14). Phagocytes (including neutrophils and monocytes) are key players in controlling rapid replicating NTS within the gut mucosa and hence in preventing the spread of NTS to systemic organs (15). Studies in both humans and mice have shown that P. falciparum-derived products such as hemozoin, heme, and heme oxygenase-1 mediate the reduction in phagocytosis and oxidative burst activities (16–18). Some studies have shown that during acute malaria, levels of the proinflammatory cytokine interleukin-12 (IL-12) are reduced whereas levels of the anti-inflammatory cytokine IL-10 are increased (19–22). The anti-inflammatory environment, coupled with reduced levels of phagocytosis and oxidative burst activities during malaria, is thought to create a favorable setting for NTS replication within the gut mucosa and bloodstream compartments. However, the role of humoral immunity to NTS during P. falciparum infection has not been explored extensively, although its role in nonmalarial children has been studied before (23–25).

Immunoglobulin G (IgG) antibodies to NTS targeting lipopolysaccharide (LPS) are thought to confer some protection against NTS bacteremia in African children (23, 25, 26). Opsonizing anti-NTS LPS IgG antibodies mediate NTS killing in a cell-free manner through the complement cascade membrane attack complex (MAC) and also facilitate killing by phagocytes, which involves phagocytosis and respiratory burst-mediated killing (24). We envisaged that exploring the role of humoral immunity to iNTS during malaria will broaden our understanding of the association between iNTS and malaria and augment the studies that were previously focused on cellular immunity. Therefore, we examined cell-free bactericidal activities and cellular bactericidal activities against NTS in a cohort of children with uncomplicated P. falciparum infections. We show that during malaria, P. falciparum infection impairs serum bactericidal immunity to *S*. Typhimurium via altered complement C3 deposition on *S*. Typhimurium in addition to the impairment of the respiratory burst of phagocytes which was known before, providing a comprehensive explanation for the increased susceptibility to iNTS seen in children during malaria.

## RESULTS

### Transient loss of serum bactericidal immunity to *S*. Typhimurium during current or convalescent P. falciparum infection.

**FIG 1:**
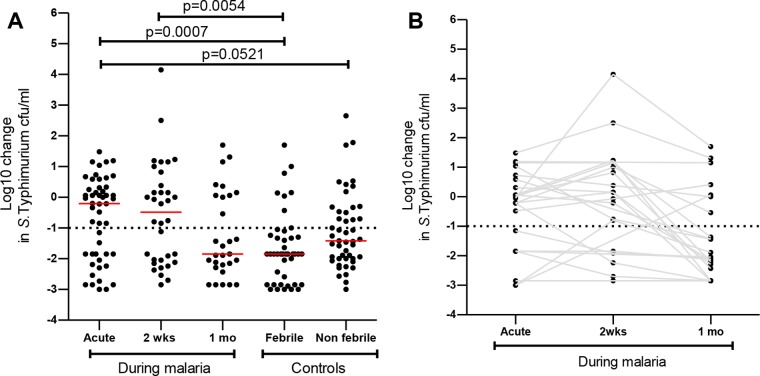
Transient loss of serum bactericidal immunity to *S*. Typhimurium during current and convalescent P. falciparum infection. Serum bactericidal activity was reported as the log10 change in *S*. Typhimurium CFU counts per milliliter from the baseline, and this was plotted as indicated during malaria and in controls. (A) Red bars represent the median, and statistical differences were determined by the Mann-Whitney U test. (B) Serum bactericidal activity measurements for each subject during current and convalescent malaria are linked by solid gray lines.

We have previously shown that acquisition of serum bactericidal activity (SBA) with respect to *S*. Typhimurium correlates with the decline in iNTS infections in childhood in individuals not infected with P. falciparum (23, 25). Therefore, we first examined SBA to determine whether SBA with respect to *S*. Typhimurium is reduced in P. falciparum-infected children. We found that SBA with respect to *S*. Typhimurium was reduced but did not reach statistical significance difference in children with acute malaria (median, −0.20 log10 [interquartile range {IQR}, −1.85, 0.32]) compared to nonfebrile malaria-negative children (median, −1.42 log10 [IQR, −2.0, −0.47], *P* = 0.052) (Fig. 1A). SBA with respect to *S*. Typhimurium was significantly reduced in children with acute malaria (median, −0.20 log10 [IQR, −1.85, 0.32], *P* = 0.0007) and at day 14 in convalescence (median, −0.49 log10 [IQR, −2.0, 0.49], *P* = 0.0054) compared to febrile malaria-negative children (median, −1.85 log10 [IQR, −2.85, −1.24]) (Fig. 1A). SBA with respect to *S*. Typhimurium at 30 days in convalescence (median, −1.85 log10 [IQR, −2.24, 0.06]) was similar to that seen with febrile malaria-negative children (median, −1.85 log10 [IQR, −2.85, −1.24], *P* = 0.43) and nonfebrile malaria-negative children (median, −1.42 log10 [IQR, −2.0, −0.47], *P* = 0.39) (Fig. 1A). Furthermore, in a subset of children we found that 6/23 (26%) had robust SBA with respect to *S*. Typhimurium (ability to kill *S*. Typhimurium by at least a −1.0 log10 change in *S*. Typhimurium CFU counts per milliliter) in the acute malaria phase compared to 16/23 (69.5%) at day 30 in convalescence (Fig. 1B). We also found that of 16 children who lacked robust SBA with respect to *S*. Typhimurium in the acute malaria phase, 10 (62.5%) attained robust SBA with respect to *S*. Typhimurium at day 30 in convalescence.

**FIG 2:**
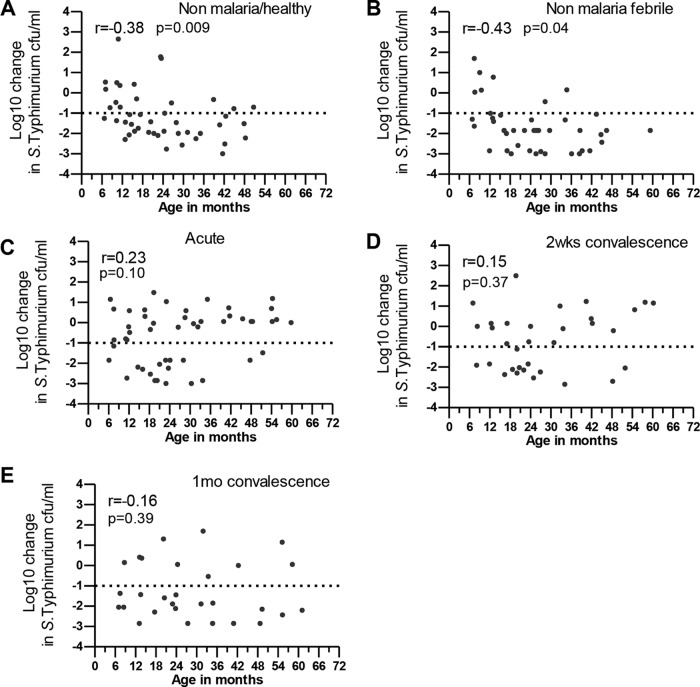
Relationship between serum bactericidal activity with respect to *S*. Typhimurium and age during malaria. Serum bactericidal activity was reported as the log10 change in *S*. Typhimurium CFU counts per milliliter from the baseline, and this was plotted against age in months as indicated in controls and during malaria. Spearman's *r* correlation coefficient and *P* values are reported.

We have previously shown that acquisition of SBA with respect to *S*. Typhimurium correlates with age in healthy children (25). We found that acquisition of robust SBA with respect to *S*. Typhimurium correlated with age development in febrile nonmalarial children and in nonfebrile malaria-negative children (Spearman's *r* = −0.43 [*P* = 0.0037] and *r* = −0.38 [*P* = 0.0086], respectively) (Fig. 2A and B). Interestingly, we observed that during acute P. falciparum infection, at day 14 and day 30 in convalescence, SBA with respect to *S*. Typhimurium did not kill *S*. Typhimurium at a level of at least a −1 log10 change in *S*. Typhimurium CFU counts per milliliter in some older children (≥24 months of age; those children were considered to have had serum results showing immunity to *S*. Typhimurium [23]) and SBA with respect to *S*. Typhimurium that poorly correlated with age (acute malaria Spearman's *r* = 0.23 [*P* = 0.11]; day 14 *r* = 0.15 [*P* = 0.37]; day 30 Spearman's *r* = −0.16 [*P* = 0.39]) (Fig. 2C to E).

**FIG 3:**
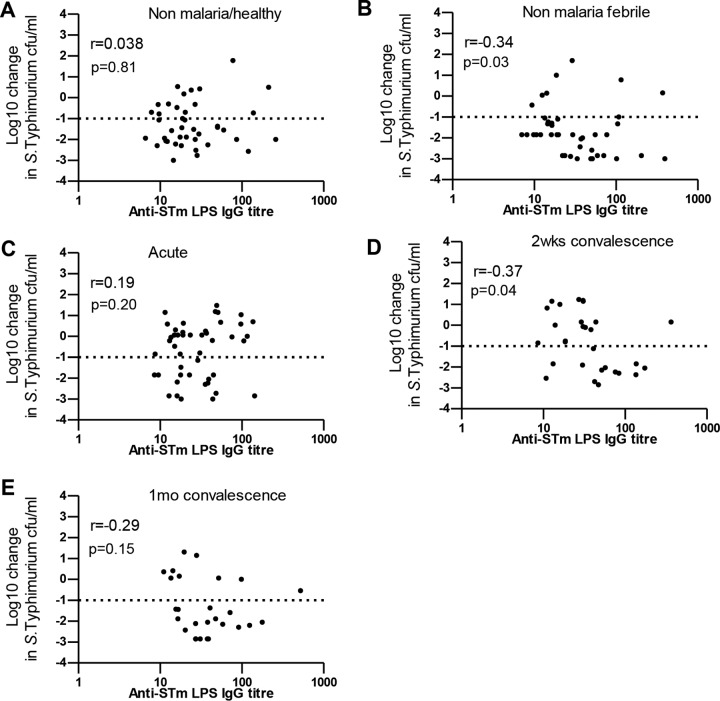
Relationship between serum bactericidal activity with respect to *S*. Typhimurium (STm) and anti-IgG antibody targeting *S*. Typhimurium LPS. Serum bactericidal activity with respect to *S*. Typhimurium was plotted for anti-IgG antibodies targeting *S*. Typhimurium LPS in controls and during malaria as indicated. Spearman's *r* correlation coefficient and *P* values are reported.

SBA with respect to *S*. Typhimurium is mainly mediated by anti-*S*. Typhimurium IgG antibodies targeting LPS (23, 25). Unexpectedly, we found that SBA with respect to *S*. Typhimurium in nonfebrile malaria-negative children poorly correlated with anti-*S*. Typhimurium LPS IgG antibody titers (Spearman's *r* = 0.038, *P* = 0.81) whereas SBA in febrile nonmalarial children correlated with anti-*S*. Typhimurium LPS IgG antibody titers (Spearman's *r* = −0.34, *P* = 0.03) (Fig. 3A and B). Interestingly, we observed that during acute malaria, SBA with respect to *S*. Typhimurium poorly correlated with anti-*S*. Typhimurium LPS IgG antibody titers (Spearman's *r* = 0.19, *P* = 0.20) whereas the correlation of SBA with anti-*S*. Typhimurium LPS IgG antibody titers was superior at day 14 and day 30 in convalescence; however, this was statistically significant only at day 14 (Spearman's *r* = −0.37 [*P* = 0.04] and *r* = −0.29 [*P* = 0.15], respectively) (Fig. 3C to E). These findings suggest that P. falciparum infection induced the transient loss of serum bactericidal activity with respect to *S*. Typhimurium in P. falciparum-infected children and that the effect was independent of age and acquired antibody immunity.

**FIG 4:**
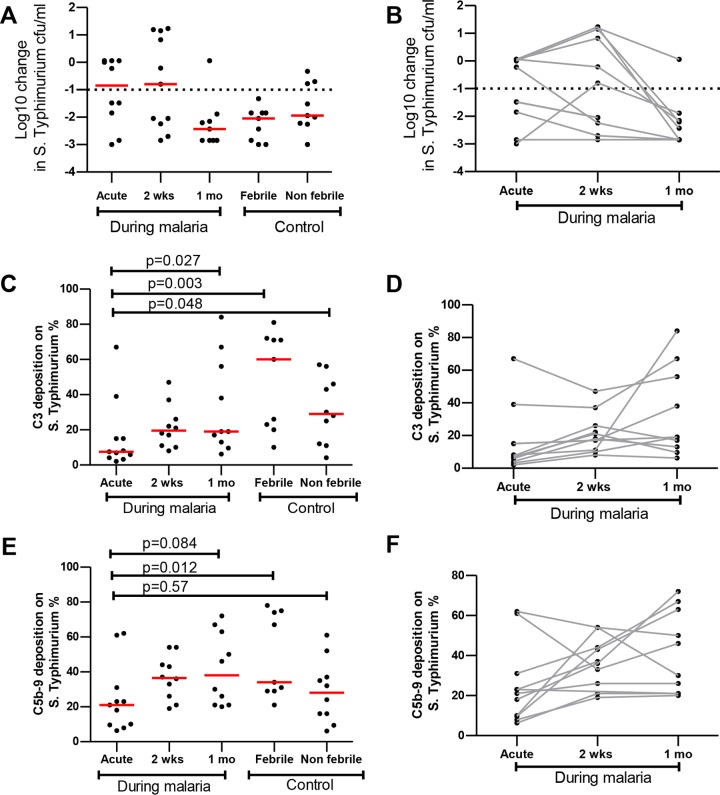
Reduced C3 deposition on *S*. Typhimurium during the acute phase of P. falciparum infection in children. Serum (*n* = 10) was randomly selected from donor children >24 months of age during malaria and from controls. (A) Serum bactericidal activity was reported as the log10 change in *S*. Typhimurium CFU counts per milliliter from the baseline, and this was plotted as indicated during malaria and in controls. (B) Serum bactericidal activity and malaria were linked. (D and F) The proportions of C3 deposition (D) and C5b-9 deposition (F) on *S*. Typhimurium measurements for each subject during current and convalescent malaria are linked by solid gray lines. Red bars represent the median, and statistical differences were determined by the Wilcoxon signed-rank test and Mann-Whitney U test.

To explore this further, we randomly selected serum samples (*n* = 10) from children (>24 months old) to examine levels of complement C3 and C5b-9 deposition during malaria (Fig. 4). Interestingly, we found that C3 deposition on *S*. Typhimurium was significantly lower in febrile P. falciparum-infected children (median, 7.5% [IQR, 4.1, 15.0]) than in febrile malaria-negative children (median, 60% [IQR, 21.5, 71.5], *P* = 0.003) and nonfebrile malaria-negative children (median, 29% [IQR, 11.8, 48.0], *P* = 0.048) (Fig. 4C and E). C3 deposition was also lower in febrile P. falciparum-infected children (median, 7.5% [IQR, 4.1, 15.0]) than at day 30 of convalescence (median, 19% [IQR, 12.1, 58.8], *P* = 0.027) and that the level was similar at day 14 in convalescence (median, 19.5% [IQR, 10.7, 28.7], *P* = 0.113) (Fig. 4C and D).

C5b-9 deposition on *S*. Typhimurium was significantly lower in febrile P. falciparum-infected children (median, 21% [IQR, 9.6, 31.0]) than in febrile malaria-negative children (median, 34% [IQR, 29, 74.5], *P* = 0.012) but was not significantly different from that seen in nonfebrile malaria-negative children (median, 28% [IQR, 14.35, 40.8], *P* = 0.57). There were no significant differences in C5b-9 deposition on *S*. Typhimurium in febrile P. falciparum-infected children (median, 21% [IQR, 9.6, 31.0]) from the levels seen at day 14 in convalescence (median, 24% [IQR, 24.8, 46.5], *P* = 0.084) and at day 30 in convalescence (median, 38% [IQR, 29, 64], *P* = 0.084) (Fig. 4E and F). Taken together, the data suggest a transient increase in consumption of the C3 complement component during acute malaria which rebounded to levels comparable to those seen with nonmalarial children by day 14 of malaria convalescence.

### Serum is a prerequisite for blood cell killing of *S*. Typhimurium.

**FIG 5:**
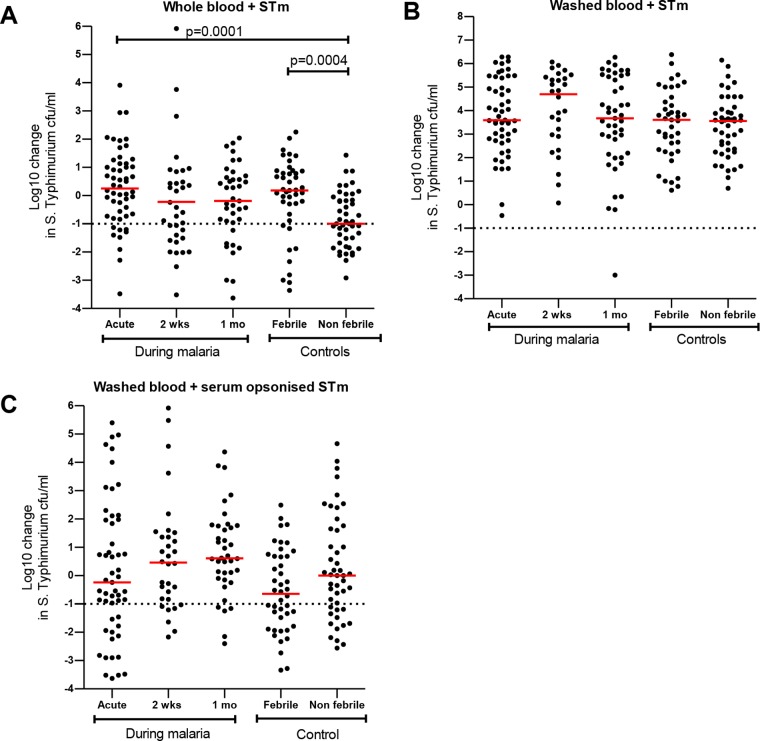
Reduced blood cell killing of *S*. Typhimurium (STm) in malarial and nonmalarial febrile children. Whole-blood (A), washed-blood (B), or serum-opsonized washed-blood (C) bactericidal activity was reported as the log10 change in *S*. Typhimurium CFU counts per milliliter from the baseline and plotted as indicated during malaria and in controls. Red bars represent the median, and statistical differences were determined by the Mann-Whitney U test.

*S*. Typhimurium is a facultative intracellular organism that requires the action of both cellular immunity and humoral immunity to be effectively controlled. We therefore examined if levels of whole-blood killing of *S*. Typhimurium were also reduced during P. falciparum infection. We found that whole-blood bactericidal activity (WBBA) with respect to *S*. Typhimurium was reduced during malaria at the acute stage (median, 0.25 log10 [IQR, −0.73, 1.13], *P* = 0.0001), day 14 in convalescence (median, −0.51 log10 [IQR, −1.53, 0.57], *P* = 0.110), and day 30 in convalescence (median, −0.19 log10 [IQR, −0.96, 0.64], *P* = 0.009) and in febrile malaria-negative children (median, 0.18 log10 [IQR, −0.66, 0.87], *P* = 0.004) compared to nonfebrile malaria-negative children (median, −1.0 log10 [IQR, −1.68, −0.16]) (Fig. 5A).

Humoral immunity enhances intracellular killing of *S*. Typhimurium (24). We observed that washed-blood-cell bactericidal activity (WBCBA) with respect to *S*. Typhimurium during malaria was abrogated in febrile malaria-negative children and nonfebrile malaria-negative children under all washed-blood-cell conditions examined (Fig. 5A and B). To determine if serum-mediated immunity is required for efficient washed-blood-cell killing of *S*. Typhimurium, we examined the assay after *S*. Typhimurium was opsonized with autologous serum, and we found that killing of *S*. Typhimurium was partially restored (Fig. 5). We show that WBBA with respect to *S*. Typhimurium was reduced during malaria and in febrile illness in children and that serum opsonization is essential for cellular killing.

### Reduced *S*. Typhimurium-specific neutrophil respiratory burst in malaria and febrile nonmalarial children.

**FIG 6:**
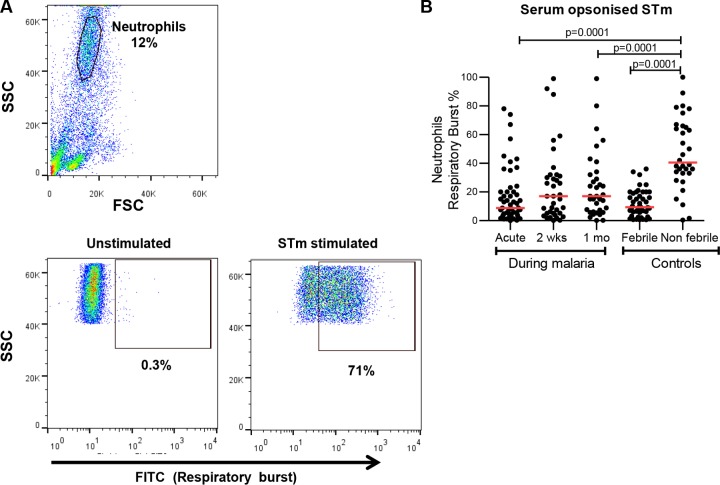
Reduced *S*. Typhimurium-specific neutrophil respiratory burst activity in malarial and nonmalarial febrile children. (A) The representative gating strategy for neutrophils using forward-scatter (FSC) and side-scatter (SSC) expression followed by neutrophil respiratory burst activity plots in unstimulated or *S*. Typhimurium-stimulated cells is shown. (B) Percentages of *S*. Typhimurium-specific neutrophil respiratory burst-positive cells during malaria and in controls were plotted as indicated. Red bars represent the median, and statistical differences were determined by the Mann-Whitney U test.

To identify the specific bactericidal function that was altered in children with malaria and febrile illness, we examined neutrophil respiratory burst activity (NRBA) as it is a key mechanism for intracellular pathogen killing. We found that NRBA was significantly reduced in children during acute malaria (median, 8.8% [IQR, 3.7, 20], *P* = 0.0001) and also in febrile malaria-negative children (median, 9.4% [IQR, 4.4, 19.5], *P* = 0.0001) compared to nonfebrile malaria-negative children (median, 40.5% [IQR, 33, 65.8]) (Fig. 6). We observed that in P. falciparum-infected children, there was a modest trend for improved respiratory burst at day 14 (median, 17% [IQR, 5.1, 31.5], *P* = 0.135) and day 30 (median, 17% [IQR, 6.1, 32], *P* = 0.042) in convalescence compared to the acute malaria phase (median, 8.8% [IQR, 3.7, 20]) (Fig. 6B) but that there remained a significant defect even at 1 month. This shows that both malarial and nonmalarial febrile children have impaired NRBA with respect to *S*. Typhimurium. The pattern over time was similar to that seen for whole-blood killing, in keeping with the oxidative burst being a dominant mechanism for the observed whole-blood bacterial killing.

## DISCUSSION

This study extended our understanding of how P. falciparum infection compromises phagocyte-dependent immunity to NTS in children (16–18) and provided an additional explanation for the observed increased susceptibility of children to iNTS in settings of malaria endemicity. Our report describes the transient loss of serum bactericidal immunity to iNTS during current or convalescent P. falciparum infection in children. This loss of serum bactericidal immunity was specific to children who were febrile from malaria compared to other causes of fever. P. falciparum infection appears to compromise serum bactericidal immunity to iNTS in older children, and the effect does not correlate with preexisting IgG antibodies to *S*. Typhimurium LPS. This is likely caused by the increased consumption of the C3 complement component during P. falciparum infection.

In this study, we demonstrated the transient loss of bactericidal humoral immunity to *S*. Typhimurium in children with current or convalescent P. falciparum infection. The loss in bactericidal SBA with respect to *S*. Typhimurium was independent of age and anti-*S*. Typhimurium LPS IgG antibody titers during acute malaria and at day 14 of convalescence, and robust SBA with respect to *S*. Typhimurium was restored after 30 days of convalescence. This was explored further by examining deposition of complement components on *S*. Typhimurium. Consistent with previous findings (27), we found that the level of deposition of mainly the C3 complement component was transiently reduced during the acute phase of P. falciparum infection and rebounded at days 14 and 30 in malaria convalescence to levels comparable to those seen with nonmalaria controls. This is in keeping with the lack of robust SBA with respect to *S*. Typhimurium seen during acute malaria in some children with high anti-*S*. Typhimurium LPS-IgG antibody titers. Increased consumption of complement components, particularly C3, during acute malaria as observed in the current study and other studies (27), which are crucial for antibody dependent-complement killing of Gram-negative bacteria (23, 28), may favor the proliferation of *S*. Typhimurium. We observed that at day 14 in malaria-convalescent children, SBA with respect to *S*. Typhimurium remained poor despite complement protein C3 on *S*. Typhimurium rebounding to normal levels, suggesting that other factors may be involved in compromising serum bactericidal immunity during malaria, and this needs to be investigated further. P. falciparum has also developed complement killing escape strategies; it is possible that serum killing is abrogated during malaria via P. falciparum recruitment of factor H protein which prevents complement cascade activation via the C3b (29–31), ultimately blocking complement-mediated NTS lysis. Furthermore, P. falciparum infection may compromise humoral immunity to NTS via a reduction of the antibody opsonization capacity as observed in some studies (32), as well as via defective complement cascade activation. This observation suggests that in children in settings where exposure to NTS is frequent and where malaria is highly endemic, humoral bactericidal immunity may be lost during repeated malaria episodes, increasing overall susceptibility to iNTS by favoring NTS proliferation and systemic infection.

We have shown that levels of WBBA with respect to *S*. Typhimurium are reduced in both P. falciparum-infected and febrile malaria-negative children. Consistent with previous observations (24), our findings indicate that serum immunity plays a crucial role in both cell-free and intracellular killing of NTS. These findings provide support for antibody-based NTS vaccine development strategies, as they are likely to elicit both extracellular and intracellular protection against iNTS. Neutrophil respiratory burst constitutes a key mechanism of intracellular effector function for Salmonella killing (33). Consistent with results of previous studies (16–18, 21), we have shown that NRBA with respect to *S*. Typhimurium is reduced in both P. falciparum-infected and febrile malaria-negative children compared to nonfebrile malaria-negative children. It has long been known that P. falciparum infection-derived products, including heme, heme oxygenase, and hemozoin, compromise neutrophil and monocyte effector functions in both humans and mice (16–18, 21, 34). In contrast to transient loss of serum killing with respect to *S*. Typhimurium, we observed that both NRBA and WBBA were reduced for a period longer than 30 days in malaria-convalescent children. This is in keeping with a previous observation (18). Surprisingly, we found that NRBA was also reduced in febrile malaria-negative children compared to nonfebrile malaria-negative children. How nonmalarial febrile illness compromises the neutrophil respiratory burst is not clear. In this study, we did not confirm the etiology of nonmalarial febrile illnesses. Identifying the causes of these febrile illnesses might provide insights into the mechanisms behind impaired neutrophil respiratory burst. We recommend further investigations into the contribution of reduced C3 levels during the acute phase of malaria to poor NRBA and WBBA with respect to *S*. Typhimurium, an aspect that was not explored in our current study. These findings suggest that the loss of intracellular killing of NTS in P. falciparum-infected and nonmalarial febrile children is likely due to impaired neutrophil respiratory burst activity.

### Conclusion.

We have demonstrated that P. falciparum infection transiently compromises humoral immunity to NTS in children, extending our knowledge of the reasons that P. falciparum infection compromises cellular immunity to NTS. The results of this study broaden our understanding of the immunologic basis of increased susceptibility to iNTS during acute or convalescent malaria and of the epidemiological association of malaria and iNTS in regions of malaria endemicity. The global immune defects induced by P. falciparum infection may render children from regions of malaria endemicity at risk of infections by not only iNTS but also other enteric Gram-negative bacteria (35). Our study results further highlight the need to improve management of concurrent malaria and IBIs, particularly by developing rapid diagnostic test for IBIs, ideally to be run in parallel with rapid diagnostic tests for malaria. This could significantly improve the identification of malaria and IBIs, promote rational prescribing of antimicrobial agents, and improve health outcomes.

## MATERIALS AND METHODS

### Recruitment of study participants and follow-up.

We recruited 154 children aged 6 to 60 months at a Community Health Centre in Blantyre, Malawi, from January 2016 to August 2016. The study participants comprised 59 febrile children presenting with uncomplicated malaria, 49 febrile malaria-negative children, and 46 nonfebrile malaria-negative children (Table 1). P. falciparum-infected children were followed up at day 14 (*n* = 42) and day 30 (*n* = 41) during convalescence. The uncomplicated-malaria group was comprised of children with acute-phase P. falciparum infection who presented to the hospital for medical care; they were febrile (>37.8°C) at the time of recruitment, had positive malaria rapid diagnostic test and positive malaria blood film results, a Blantyre coma score of 5 (36), a hemoglobin (Hb) level of >5 g/dl, and a serum glucose level of ≥45 mg/dl. Children with a positive HIV antibody test result, severe anemia (Hb, ≤5 g/dl), malnutrition (weight-for-height Z-score, less than −2), or other chronic illness were excluded from the study. A 3-ml venous blood sample was collected from each participant at recruitment and during follow-up. Participants presenting with uncomplicated malaria were treated according to Malawi Government guidelines, before blood sample collection. Ethical approval for the study was obtained from the College of Medicine Research Ethics Committee (protocol number P.08/15/1785), and written informed consent was obtained from parents or guardians of participating children.

**TABLE 1:** Study participants' demographic and clinical features

Parameter	Values				
	During malaria			Nonmalarial controls	
	Acute (*n* = 59)	2 wks (*n* = 42)	1 mo (*n* = 41)	Febrile (*n* = 49)	Nonfebrile (*n* = 46)
No. (%) of female participants	34 (58)	26 (62)	25 (61)	27 (55)	15 (39)
Median age in mo (range)	22.8 (6–59.8)	23.5 (6.6–60.2)	24.1 (7–61)	22.8 (6–59.3)	21.9 (6.7–50.7)
Median wt in kg (range)	10.2 (6.9–17)	10.5 (7 −16.1)	10.1 (7.1–16.3)	10.1 (6.9–15.4)	10.5 (7.1–15.5)
Median ht in cm (range)	84 (66–113)	85 (75–113)	85 (75.6–113)	84.5 (74–104)	82.5 (64–106)
Median MUAC^*a*^ in cm (range)	14 (10.2–19)	13.8 (10–18.7)	13.95 (10.4–18.8)	13.4 (10–16.2)	14.2 (10.2–17.5)
Median Hb level in g/dl (range)	9.5 (5.9–12.3)	9.7 (7.3–12.4)	10.3 (8.4–12.7)	10.8 (7.9–18.7)	11 (5.2–13.6)
Median absolute no. of lymphocytes × 10^3^/μl (range)	3.4 (0.9–9.8)	5.3 (2.2–9.2)	5.3 (1.7–15.2)	4.2 (1.2–14.9)	5.4 (2.2–69.2)
Median absolute no. of neutrophils × 10^3^/μl (range)	3.7 (0.93–11.4)	2.8 (1.2–3.9)	2.8 (0.5–5.9)	3.6 (0.2–19.9)	2.3 (0.18–22.2)
No. (%) of subjects with:					
Splenomegaly	20 (39)	1 (2.4)	0 (0)	4 (8.2)	1 (2.6)
Cough	24 (40.6)	9 (21.4)	9 (22)	29 (59)	0 (0)
Shortness of breath	1 (1.7)	0 (0)	0 (0)	13 (26.5)	0 (0)
Vomit	21 (35.6)	1 (2.4)	2 (4.9)	25 (51)	0 (0)
Diarrhea	14 (23.7)	0 (0)	2 (4.9)	14 (28.6)	0 (0)

a MUAC, mid-upper arm circumference.

### Quantification of *S*. Typhimurium-specific SBA.

Serum bactericidal activity (SBA) assays were performed as previously described (23). Briefly, serum or phosphate-buffered saline (PBS) was mixed with *S*. Typhimurium D23580 (37), adjusted to 1.0 × 10^6^ CFU/ml, and incubated at 37°C for 180 min. Test samples were serially diluted and plated in triplicate on Luria Bertani agar. Colony counts of *S*. Typhimurium were done after 24 h of incubation. Log10 changes in *S*. Typhimurium CFU counts per milliliter from the baseline were reported.

### Quantification of *S*. Typhimurium-specific whole-blood-cell and washed-blood-cell killing.

Three conditions were prepared as previously described (24). For condition 1, whole blood was used in a whole-blood bactericidal assay (WBBA). For condition 2, whole blood was washed twice with RPMI 1640 at 1,000 rpm for 10 min before being used in a washed-blood-cell bactericidal assay (WBCBA). For condition 3, *S*. Typhimurium adjusted to 1.0 × 10^7^ CFU/ml was first opsonized with 1:10 serum from each participating child for 20 min at room temperature (RT) before challenging washed blood cells in a washed-blood-cell and serum-opsonized assay (WBCSOA). All conditions were challenged with *S*. Typhimurium adjusted to a final concentration of 1.0 × 10^7^ CFU/ml. Colony counts were performed as described for the SBA experiment above.

### Quantification of anti-*S*. Typhimurium IgG antibody titer by ELISA.

These experiments were performed as previously described (25). Briefly, enzyme-linked immunosorbent assay (ELISA) plates (Nunc-Immuno) were coated overnight with 100 μl of carbonate-bicarbonate buffer (Sigma-Aldrich) per well containing 7.5 μg/ml *S*. Typhimurium LPS antigen (Alexis Biochemicals). Plates were washed with PBS containing 0.05% Tween 20 and blocked with 200 μl/well blocking buffer (PBS–1% bovine serum albumin [BSA]) for 1 h at 37°C. Test serum prepared at 1:20 in dilution buffer (PBS–0.05% Tween 20–1% BSA) was serially diluted 3-fold and incubated at 37°C for 1 h. After washing, 100 μl of 1:2,000 secondary goat anti-human IgG-AP antibodies (Southern Biotech) was added and incubated for 1 h at 37°C. Finally, after washing, 100 μl of SigmaFAST p-nitrophenyl phosphate substrate was added to each plate and the plate was read after 30 min using a BioTek ELx800 reader (BioTek Instruments, USA) at 405 nm.

### Quantification of complement components binding on the surface of *S*. Typhimurium.

These experiments were performed as previously described (23, 28). A 5-μl volume of *S*. Typhimurium was gently mixed at 2 × 10^8^ CFU/ml with 45 μl of undiluted serum or PBS (control) at RT for 20 min. Samples were washed twice with 1 ml PBS by spinning for 5 min at 3,300 × *g*. A 2-μl volume of anti-C3c fluorescein isothiocyanate (FITC)-conjugated antibody (Abcam) was added to 50 μl of pellet to measure C3 deposition. A 1-μl volume of anti-C5b-9 neo-epitope antibody (Abcam) and 2 μl of rabbit anti-mouse FITC-conjugated antibody (Abcam) were added to 50 μl of pellet to measure MAC levels. Samples were washed twice with 1 ml of PBS after 20 min of incubation at RT and fixed with 200 μl of 1% formaldehyde–PBS. Samples were acquired on a CyAN ADP flow cytometer (Beckman Coulter) and analyzed using Flow Jo version 7.6.5.

### Quantification of neutrophil respiratory burst.

A Phagoburst test kit (Glycotope Biotechnology) was modified to measure neutrophil respiratory burst levels as previously described (24). Whole blood (45 μl) was incubated on ice for 10 min and then stimulated with serum-opsonized *S*. Typhimurium at 1.0 × 10^8^ CFU/ml or with a wash solution containing instalmed-salts (control). Samples were then incubated for 10 min at 37°C to allow phagocytosis followed by 10 min of incubation at 37°C after addition of dihydrorhodamine 123 to promote oxidation. The reaction was stopped by application of 1:10 lysing solution for 20 min at RT. Samples were acquired on CyAN ADP flow cytometer (Beckman Coulter) and analyzed using Flow Jo version 7.6.5.

### Statistical analyses.

Statistical analyses were performed using GraphPad Prism version 5 (GraphPad Software, USA). Log10 change in bactericidal activity with respect to *S*. Typhimurium, percentage of neutrophil respiratory burst-positive cells, and complement deposition were examined for normality of distribution using the D'Agostino and Pearson omnibus normality test. Nonparametric data were compared using the Mann-Whitney U test or the Wilcoxon signed-rank test for paired *t* tests. Median and interquartile range (IQR) were reported, and a *P* value of less than 0.05 was considered statistically significant. Spearman's correlation coefficient *r* was used to determine relationships between bactericidal activity and age and anti-*S*. Typhimurium LPS IgG antibody titers during malaria.
